# Rapid detection of porcine circovirus type 4 *via* multienzyme isothermal rapid amplification

**DOI:** 10.3389/fvets.2022.949172

**Published:** 2022-07-28

**Authors:** Yuqing Li, Yanli Zhao, Chen Li, Kankan Yang, Zhe Li, Wenbin Shang, Xiangjun Song, Ying Shao, Kezong Qi, Jian Tu

**Affiliations:** ^1^Anhui Province Engineering Laboratory for Animal Food Quality and Bio-safety, College of Animal Science and Technology, Anhui Agricultural University, Hefei, China; ^2^Anhui Province Key Laboratory of Veterinary Pathobiology and Disease Control, Anhui Agricultural University, Hefei, China

**Keywords:** porcine circovirus type 4, multienzyme isothermal rapid amplification, rapid detection, clinical samples, specificity, sensitivity

## Abstract

Porcine circovirus type 4 (PCV4) is a newly emerging pathogen that was first detected in 2019 and is associated with diverse clinical signs, including respiratory and gastrointestinal distress, dermatitis and various systemic inflammations. It was necessary to develop a sensitive and specific diagnostic method to detect PCV4 in clinical samples, so in this study, a multienzyme isothermal rapid amplification (MIRA) assay was developed for the rapid detection of PCV4 and evaluated for sensitivity, specificity and applicability. It was used to detect the conserved Cap gene of PCV4, operated at 41°C and completed in 20 min. With the screening of MIRA primer-probe combination, it could detect as low as 10^1^ copies of PCV4 DNA per reaction and was highly specific, with no cross-reaction with other pathogens. Further assessment with clinical samples showed that the developed MIRA assay had good correlation with real-time polymerase chain reaction assay for the detection of PCV4. The developed MIRA assay will be a valuable tool for the detection of the novel PCV4 in clinical samples due to its high sensitivity and specificity, simplicity of operation and short testing time.

## Introduction

Porcine circovirus (PCV) is the smallest known virus and is classified as a member of family *Circoviridae*, genus *Circovirus* (1). Four species of PCV were named, respectively, in the order of discovery as porcine circovirus type 1 (PCV1), porcine circovirus type 2 (PCV2), porcine circovirus type 3 (PCV3), and the recently reported porcine circovirus type 4 (PCV4) (2, 3). The first PCV1 was shown to be nonpathogenic to pigs (4) and PCV2 could cause porcine dermatitis and nephropathy syndrome (PDNS), postweaning multisystemic wasting syndrome (PMWS) and congenital tremors (5). Using metagenomic sequencing, Palinski discovered PCV3 in 2016 (2), which proved to be pathogenic, with PDNS-like disease in the experimental of the healthy piglets within 8 weeks old (6). In 2019, a novel porcine circovirus, designated as PCV4 was first reported in China, discovered in pigs suffering from respiratory, enteric and PDNS signs and from healthy pigs (7). The size of the PCV4 genome was 1,770 nucleotides, like PCV2 and PCV3, and contained two major ORFs, ORF1, and ORF2, which encoded separate replicase (Rep) and capsid (Cap) proteins (7). Two major genes were: a Rep gene spanning 891nt and a Cap gene spanning 687nt (7). The virus PCV4 showed the highest genomic identity to mink circovirus at 66.9% and had identities of 43.2 to 51.5% to the other PCV genomes (7).

To date, PCV4 infections have been reported in Korea and identified in Henan, Shanxi, Jiangsu, Inner Mongolia and Guangxi provinces of China (7–10). To better investigate the epidemiology of PCV4, it was necessary to develop a sensitive and specific detection method. Methods used to detect PCV4 include loop-mediated isothermal amplification (LAMP) (11, 12), enzyme-linked immunosorbent assay (ELISA) (13) and real-time polymerase chain reaction (PCR) (14, 15). However, LAMP technology is time-consuming and requires a large set of six primers and high temperatures. The repeatability of ELISA is poor and there are multiple interfering factors, particularly temperature and time. Real-time PCR assays are time-consuming, so the development of a simple, rapid and cost-effective assay with high specificity and sensitivity is essential for detecting PCV4.

**Figure 1:**
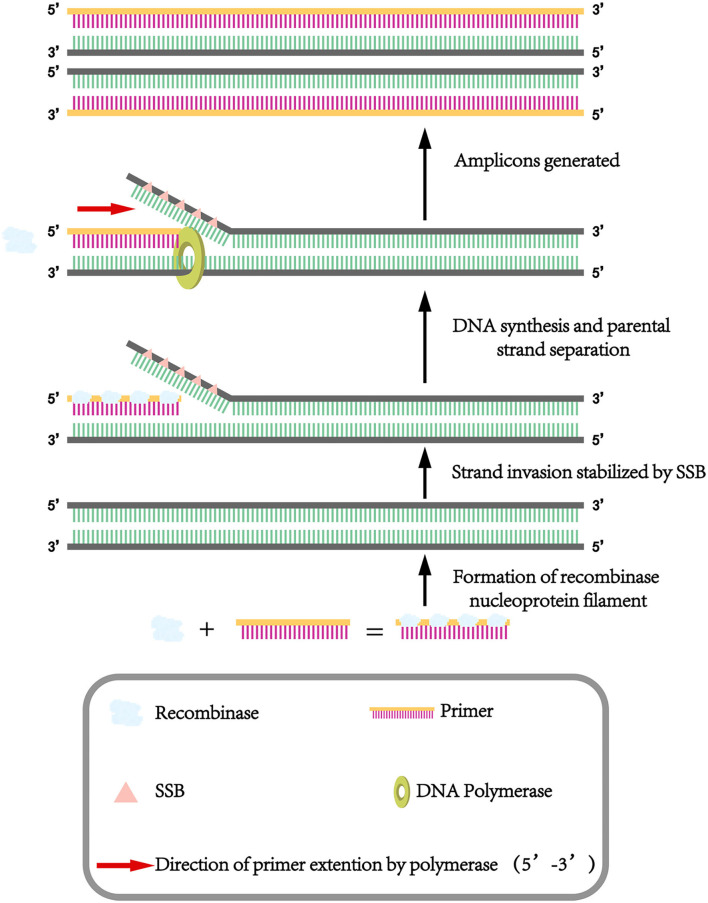
Schematic diagram of the MIRA.

Isothermal nucleic acid amplification technology has developed rapidly in recent years, which is performed at a constant temperature for 20 min, so a thermal cycler is unnecessary and this makes it a convenient and rapid tool for pathogen detection. Multienzyme isothermal rapid amplification (MIRA) is a modified version of recombinase polymerase amplification (RPA), belonging to isothermal nucleic acid amplification techniques that can be conducted at constant temperatures without the need for thermocycles, as seen in Figure 1. With the help of the auxiliary protein and single-stranded DNA-binding protein (SSB), the recombinase and the primer form the D-loop region and when the target region is complementary to the primer, the recombinase deviates while the polymerase binds to the 3′ end of the primer and starts the chain extension, quickly completing the amplification of the target fragment at 37°C to 42°C for 20 min. In comparison to LAMP, ELISA and real-time PCR, MIRA is faster and simpler to run, requiring only a pair of primers, a lower temperature at 37°C to 42°C and a shorter run time under 20 min. To the best of the authors knowledge, the detection of PCV4 by MIRA has not been reported, so it was developed in this study.

## Materials and methods

### Virus strains

The viruses PCV2, PCV3, PCV4, porcine parvovirus (PPV) and the vaccine strains of porcine reproductive and respiratory syndrome virus (PRRSV, R98 strain), pseudorabies virus (PRV, HB-98 strain), and classical swine fever virus (CSFV, AV1412 strain) were used in this study. PCV2, PCV3, PCV4, and PPV were stored in the Anhui Province Key Laboratory of Veterinary Pathobiology and Disease Control. Porcine reproductive and respiratory syndrome virus (PRRSV, R98 strain), pseudorabies virus (PRV, HB-98 strain), and classical swine fever virus (CSFV, AV1412 strain) were purchased from China Animal Husbandry Industry Co., LTD (Beijing, China).

### Nucleic acid extraction

The viral DNA of PCV2, PCV3, PPV, and PRV were extracted using a DNA extraction kit (Tiangen Bio-Tech, Beijing, China). The viral RNA of PRRSV and CSFV were extracted using an RNA extraction kit (Tiangen Bio-Tech, Beijing, China) and the obtained RNA was reverse transcribed into cDNA using a PrimeScript™ RT Master Mix (Takara Biotechnology, Dalian, China), all following the manufacturer's instructions. All DNA and cDNA templates were stored at −20°C until the assays were performed.

### Generation of standard plasmids

The complete Cap gene of PCV4 was amplified with Cap-forward and Cap-reverse primers, as seen in Table 1. It was then cloned into the vector pMD-19T (TaKaRa) and designated as pMD-19T-Cap. The recombinant plasmids were sequenced by Nanjing Tsingke Biotechnology Co., LTD and the concentration determined using an ND-2000c spectrophotometer (NanoDrop, Wilmington, USA). The copy number of pMD-19T-Cap was calculated as the number of plasmids in copies/μL = 6.02 × 10^23^ × plasmid concentration (ng/μL)/genome length (bp) × 10^9^ × 660, then serial 10-fold dilutions from 1 × 10^5^ to 1 × 10^1^ copies/μL were created and stored at −20°C for subsequent assays.

**Table 1:** Primers and probe sequences of PCV4 used in this study.

**Named**	**Sequence (5**′**-3**′**)**	**Position**	**Source**
Cap-forward	TTATCCCTGTTTGGGGTAGTTAACAAGGT		This study
Cap-reverse	ATGCCAATCAGATCTAGGTACAG		This study
PCV4-MIRA-F1	TCCTGGGGTTTTGGAGTGAAATAGCGACTG	1255–1284	This study
PCV4-MIRA-R1	CATGAGGGAGGTGACTCTCAGCGTGTCAAG	1573–1602	This study
PCV4-MIRA-F2	TTGTTAGGCTGGAAGTGGAGGGTGTGGGTT	1213–1242	This study
PCV4-MIRA-R2	GCTTCATGAGGGAGGTGACTCTCAGCGTGT	1577–1606	This study
PCV4-MIRA-F3	TGCTGCTGTGGTTTGCCAGGACATCATAAGT	1313–1343	This study
PCV4-MIRA-R3	CCAGTAGGCGGAGATACCGGTGGAGAAGGAA	1627–1657	This study
PCV4-MIRA-F4	TTTGTTGTTAGGCTGGAAGTGGAGGGTGT	1209–1237	This study
PCV4-MIRA-R4	AACTGAAGGACTTTATCCCAAAAGGACCG	1503–1531	This study
PCV4-MIRA-F5	AAGTGGAGGGTGTGGGTTTCCCCRGAGGGGTC	1225–1256	This study
PCV4-MIRA-R5	CAAAGTCGAATTTCTGCCACTAAATGGCATTAACA	1412–1446	This study
PCV4-probe	CCACATAGTCTCCATCCAGTTGTATAGCAG[FAM-dT] [THF]C[BHQ1-dT]AGAGTAAGTCCTATT[C3spacer]	1361–1409	This study

### MIRA probe and primers

**Figure 2:**
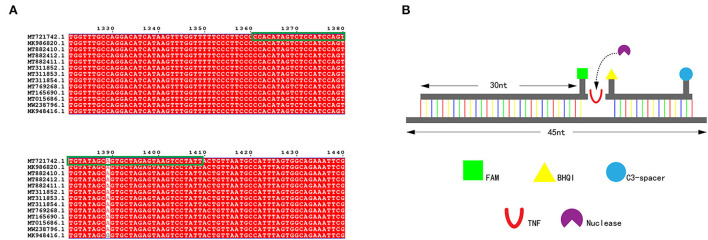
**(A)** Alignment of probe designed from partial of gene sequences of PCV4. **(B)** The schematic diagram of the specific MIRA probe.

The PCV4 sequences with accession numbers MT721742, MK98682, MT882410, MT882412, MT882411, MT311852, MT311853, MT311854, MT769268, MT165690, MT015686, MW238796 and MK948416 were aligned, as shown in Figure 2A. A specific MIRA probe was designed first as in Figure 2B, then five primer pairs were designed, targeting the highly conserved region of the Cap gene. All gene sequences used in this study were retrieved from GenBank. All primers and probes were synthesized by General Biological (Anhui, China) and the sequence of all the probes and primers are summarized in Table 1.

### Multienzyme isothermal rapid amplification

The MIRA assay was performed using a DNA constant temperature rapid amplification kit (Weifang Amp-Future Biotech, Shandong, China). The reaction system was 14.7 μL of buffer A, 5.75 μL of ddH_2_O, 1 μL of template, a 0.3 μL of 10 μmol/L probe and 1 μL of each 10 μmol/L primer in a reaction tube containing lyophilized enzyme pellets, to which 1.25 μL of buffer B was added for a total volume of 25 μL. The samples were placed in a multi-functional fluorescence quantitative polymerase chain reaction (qPCR) system (Applied Biosystems, Foster City, USA) immediately and a fluorescent signal collected every 30 s for 20 min at 41°C.

### Specificity and sensitivity of the MIRA assay

To evaluate the specificity of the MIRA assay, the MIRA reaction was performed using nucleic acid extracted from PCV4, six other porcine viruses PCV2, PCV3, PPV, PRRSV, PRV, CSFV and RNase-free distilled water (ddH_2_O). The limit of detection (LOD) of the MIRA assay for PCV4 was determined using a series of 10-fold dilutions from 10^5^ to 10^1^ copies/μL of the standard PCV4 Cap DNAs. The LOD of the MIRA assay was then compared with those of the real-time PCR using the same DNA templates described above. The amplification products were also observed under UV light.

### Real-time PCR

The real-time PCR assay was performed on a Vii A7+QuantStudio™ 3D instrument (Applied Biosystems, Foster City, USA) using the AceQ^®^ qPCR SYBR Green Master Mix (Vazyme Biotech, Nanjing, China). The forward primer was PCV4-F (5′-CCTCCACTTCCAGCCTAACA-3′), the reverse primer was PCV4-R (5′-GTCCACACCTGCACAAAGTT-3′), which were designed based on the cap gene of PCV4 (16). The conditions for the SYBR Green-based real-time PCR assay were performed in a 20 μL reaction mixture that contained 10 μL of 2 × AceQ qPCR SYBR Green Master Mix, 0.4 μL of 10 μmol/L of each primer PCV4-F and PCV4-R, 8 μL of ddH_2_O and 1.2 μL of DNA template. The negative control contained RNase-free ddH_2_O and PCV4 plasmids with 1 × 10^5^ to 1 × 10^1^ copies/μL were used as positive controls in the experiments. The reaction conditions were 95°C for 5 min, followed by 40 cycles of 95°C for 10 s and 60°C for 30 s. The melting procedure was 95°C for 15 s, 60°C for 1 min, then 95°C for 15 s.

### Clinical sample detection

After determining the sensitivity and specificity of the assay, clinical samples were evaluated and compared with results from the corresponding real-time PCR assay. Forty-seven clinical samples of lymph nodes, liver, and spleen were gathered from an abattoir suspected to be infected with porcine circovirus in Anhui Province (China) and donated by Anhui Animal Disease Prevention and Control Center. Each tissue sample was weighed at approximately 1 g, added to 1 mL of phosphate-buffered saline (PBS), homogenized in a mortar, and then transferred to a sterile centrifuge tube and centrifuged at 8,500 rpm/min for 3 min to extract nucleic acids according to the instructions of the viral genomic DNA/RNA extraction kit (Tiangen Bio-Tech, Beijing, China). The obtained liquid was viral DNA/RNA, which was stored at −80°C until used to evaluate the performance of the MIRA assay. For comparison, all the clinical samples were evaluated by real-time PCR. The amplification conditions were as previously described.

## Results

### Screening of optimal MIRA primers

**Figure 3:**
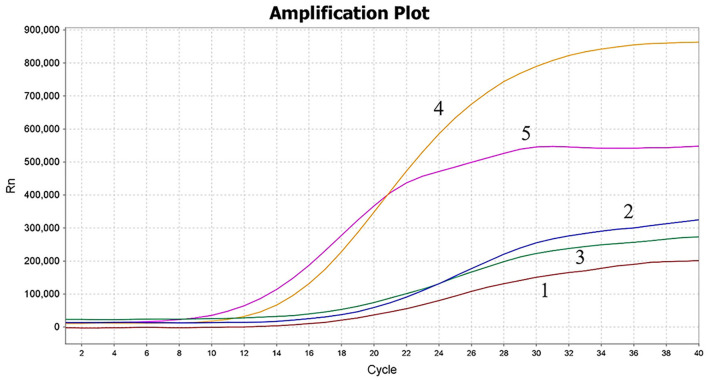
Screening of MIRA primer pairs. The amplification results of screening five primer pairs. The fifth primer set was used for the MIRA assay. 1–5: the first primer set to the fifth primer set.

In this study, five primer pairs were designed and subjected to the MIRA assay, as shown in Table 1. As revealed by the results, all five primers could recognize and amplify PCV4, while a significant difference was observed during the amplification. Compared with the other three primer pairs, the fourth primer pair generated the best amplification, but the fifth primer pair amplified within 6 min and the amplification efficiency was higher, as seen in Figure 3. Thus, the fifth primer pair was optimal and was chosen for subsequent MIRA assays.

### Specificity of the MIRA assay

**Figure 4:**
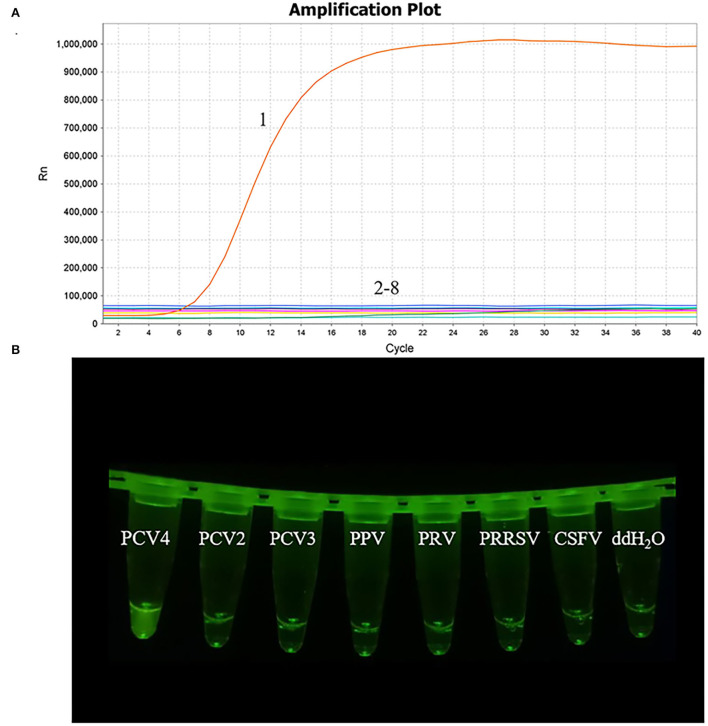
Analytical specificity result of the MIRA assay. **(A)** The amplification results of MIRA in the form of fluorescence read-out. 1, PCV4; 2, PCV2; 3, PCV3; 4, Porcine parvovirus (PPV); 5, Pseudorabies virus strain HB-98 (PRV); 6, the vaccine strains of Porcine reproductive and respiratory syndrome virus strain R98 (PRRSV); 7, Classical swine fever virus strain AV1412 (CSFV); 8, RNase-free ddH_2_O. **(B)** Direct visual observation under UV light of the amplification products of MIRA. Tubes, from left to right, nucleic acid templates corresponding to PCV4, PCV2, PCV3, PPV, PRV, PRRSV, CSFV and negative control of RNase-free ddH_2_O, respectively.

The nucleic acid of PCV2, PCV3, PPV, PRRSV, PRV, and CSFV was examined in the MIRA assay. The PCV4 samples and nuclease-free water were used as positive and negative controls, respectively, to verify the specificity of MIRA. The PCV4 produced the fluorescence signals in 4 min and PCV2, PCV3, PPV, PRRSV, PRV, and CSFV were not detected, as seen in Figure 4, which showed the developed MIRA assay had high specificity.

### Comparative sensitivity of the MIRA assay

**Figure 5:**
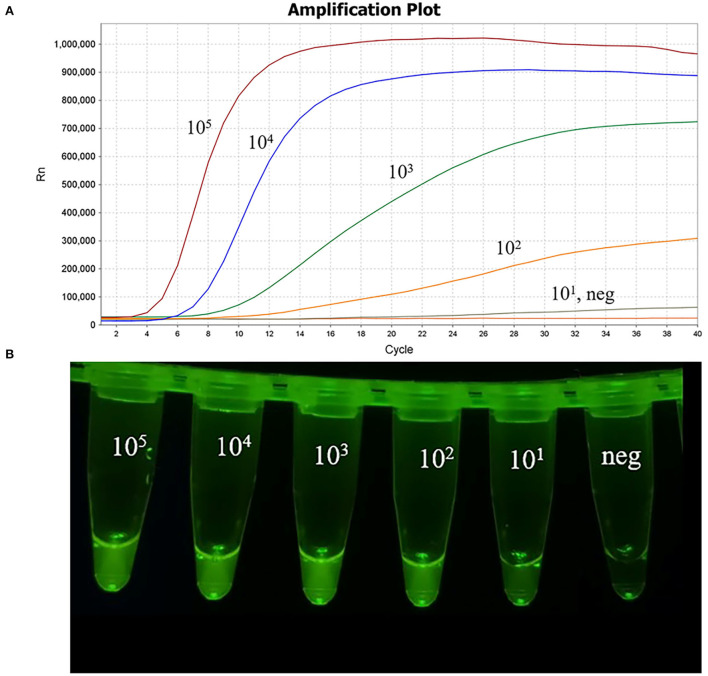
Sensitivity of the MIRA assay. The 10-fold dilutions of plasmid pMD-19T-Cap as the template for the MIRA assay. **(A)** The amplification results of MIRA combined with fluorescence read-out. Curves, 10^5^ to 10^1^ copies/μL of PCV4 plasmid DNA and neg, negative control of RNase-free ddH_2_O. **(B)** Direct visual observation under UV light of the amplification products of MIRA. Tubes, from left to right, nucleic acid templates corresponding to 10^5^ to 10^1^ copies/μL of PCV4 plasmid DNA; neg, negative control of RNase-free ddH_2_O, respectively.

The plasmid pMD-19T-Cap, ranging from 10^5^ to 10^1^ copies/μL, was used to evaluate the detection limit of MIRA and comparative with the real-time PCR assay, as previously described (17). The curves of 10^1^ copies/μL plasmid with low fluorescence value are shown in Figure 5 and the data showed that the detection limit of the MIRA assay was 10^1^ copies/μL, which was the same as the real-time PCR assay (data not shown). The sensitivity of the MIRA assay was comparable to the sensitivity of the real-time PCR assay. These results reveal that the developed MIRA assay has a high sensitivity that is suitable for PCV4 detection.

### Reproducibility of the MIRA assay

The reproducibility of MIRA was analyzed using the concentrations 10^5^ to 10^3^ copies/μL of the recombinant plasmid pMD-19T-Cap as templates and the coefficient of variation (CVs) were calculated and evaluated in triplicate at the same time or in three independent runs at separate times. The CVs of intra- and inter-assays ranged from 1.47 to 2.64% and 2.23 to 2.86% (data not shown).

### Diagnostic performance of the MIRA assay for clinical samples

To further evaluate the practicality of MIRA, 47 clinical DNA samples were performed of the MIRA and real-time PCR assays. Among these clinical samples, 3 samples were detected as PCV4-positive by both MIRA and real-time PCR, and the positive rate was 6.4% by MIRA. And the PCR products of the 3 samples were sequenced by Nanjing Tsingke Biotechnology Co., LTD, which confirmed the 3 samples were true PCV4-positive. The data were 100% in agreement with the real-time PCR results, suggesting that the MIRA could be a reliable tool to detect clinical samples of PCV4, as seen in Table 2. The results showed a high degree of agreement between the two methods for the detection of clinical samples, but MIRA was faster, indicating the potential of MIRA for clinical diagnosis.

**Table 2:** Comparison of real-time PCR and MIRA results for clinical samples.

**Result**		**MIRA**		**Total**	**Coincidence** **rate**
			**Positive**	**Negative**	
qPCR	Positive	3	0	3	100%
	Negative	0	44	44	
	Total	3	44	47	

## Discussion

The virus PCV4, a unique novel circovirus found in clinical diseases of pigs, including respiratory symptoms, intestinal symptoms, and PDNS (7) as well as in healthy pigs (11), is a potential threat to the livestock industry. Co-infection with PCV2, PCV3, and PCV4 was frequently observed in clinical samples (11), and so a sensitive and specific diagnostic method for rapid and simple detection of PCV4 infection was needed. Since the first identification of PCV4 in pigs, several PCR-based assays and isothermal DNA amplification methods such as SYBR Green-based qPCR, TaqMan probe-based qPCR, LAMP, and ELISA have been developed for PCV4 detection. These existing assays are expensive, time-consuming and complex, so the development of a new simple and rapid assay with desirable specificity and sensitivity would be an advantage for the detection of PCV4.

In this study, a MIRA method was developed based on an appropriate primer-probe combination for the rapid and sensitive detection of PCV4, which is essential for the establishment of the MIRA assay (18). In this study, five primer pairs were designed around a probe. Initially, the fourth primer pair was selected for the MIRA assay, but the detection limit was 10^3^ copies/μL. The fifth primer pair was selected and the detection limit was 10^1^ copies/μL, showing high sensitivity. The result showed that an optimal primer-probe combination should also be combined with a sensitivity assay. With MIRA, only PCV4 among multiple viruses evaluated was amplified, demonstrating the high specificity of this assay, as seen in Figure 4. Using the PCV4 plasmid DNA as a template, PCV4 was detected within 20 min for 10^5^ to 10^1^ copies/μL in Figure 5. The limit of detection of the MIRA assay was 10^1^ copies/μL, which was comparable of the TaqMan real-time PCR (19). Further validation of this method was performed using clinical samples. The detection rate of the MIRA assay was comparable of that of the real-time PCR, while the former assay was much faster than the latter. The MIRA assay detected the positive samples within 20 min. While the PCV4 detection results by MIRA could be obtained in about 20 min, the reaction time for positive samples reached up to 1 h with real-time PCR. The clinical performance of the MIRA assay was determined by evaluating the 47 clinical samples of lymph nodes, liver and spleen from a slaughterhouse in Anhui Province in China. The results showed that 3 samples were positive for PCV4 using MIRA. The same results were obtained for the real-time PCR assay, indicating that the MIRA assay was sufficient for the clinical diagnosis of PCV4 in distinct types of samples, as shown in Table 2.

In this study, the MIRA method can finish within 20 min at the temperature of 41°C. Moreover, the reagents in the lyophilized pellet are easily stored and transported. The proposed MIRA method offers rapid reaction and high specificity and sensitivity, making it a reliable alternative tool for rapid detection of PCV4.

## Data availability statement

The original contributions presented in the study are included in the article/supplementary material, further inquiries can be directed to the corresponding author/s.

## Author contributions

YL wrote and revised the manuscript. YZ performed the experiments. CL and KY assisted in manuscript writing. ZL and WS performed part of the experiments. XS, YS, and KQ provided funds and resource assistance. JT designed the experiments and provided funds. All authors contributed to the article and approved the submitted version.

## Funding

This work was supported financially by Application of supporting technology and poverty alleviation demonstration of ecological breeding of native black pig in contiguous poverty-stricken area of Dabie Mountains, Anhui (201907d06020016), 2020 University Excellent Talents Support Program (gxyqZD2020009), and Anhui University Collaborative Innovation Project (GXXT-2019-035).

## Conflict of interest

The authors declare that the research was conducted in the absence of any commercial or financial relationships that could be construed as a potential conflict of interest.

## Publisher's note

All claims expressed in this article are solely those of the authors and do not necessarily represent those of their affiliated organizations, or those of the publisher, the editors and the reviewers. Any product that may be evaluated in this article, or claim that may be made by its manufacturer, is not guaranteed or endorsed by the publisher.
